# Design and Development of a Virtual Reality-Based Mobility Training Game for People With Parkinson's Disease

**DOI:** 10.3389/fneur.2020.577713

**Published:** 2021-01-15

**Authors:** James M. Finley, Marientina Gotsis, Vangelis Lympouridis, Shreya Jain, Aram Kim, Beth E. Fisher

**Affiliations:** ^1^Division of Biokinesiology and Physical Therapy, University of Southern California, Los Angeles, CA, United States; ^2^Neuroscience Graduate Program, University of Southern California, Los Angeles, CA, United States; ^3^School of Cinematic Arts, University of Southern California, Los Angeles, CA, United States; ^4^Department of Computer Science, University of Southern California, Los Angeles, CA, United States

**Keywords:** virtual reality, walking, Parkinson's disease, cognition, user-centered design

## Abstract

People with Parkinson's disease (PD) commonly have gait impairments that reduce their ability to walk safely in the community. These impairments are characterized, in part, by a compromised ability to turn and negotiate both predictable and unpredictable environments. Here, we describe the development and usability assessment of a virtual reality training application, *Wordplay VR*, that allows people with PD to practice skills such as turning, obstacle avoidance, and problem-solving during over-ground walking in a game-based setting. Nine people with PD completed three sessions with *Wordplay VR*, and each session was directed by their personal physical therapist. Our outcome measures included perceived sense of presence measured using the International Test Commission–Sense of Presence Inventory (ITC-SOPI), levels of motivation using the Intrinsic Motivation Inventory (IMI), overall system usability using the System Usability Scale (SUS), and setup time by the physical therapists. Both the people with PD and the physical therapists rated their sense of presence in the training system positively. The system received high ratings on the interest and value subscales of the IMI, and the system was also rated highly on usability, from the perspective of both the patient during gameplay and the therapist while controlling the experience. These preliminary results suggest that the application and task design yielded an experience that was motivating and user-friendly for both groups. Lastly, with repeated practice over multiple sessions, therapists were able to reduce the time required to help their patients don the headset and sensors and begin the training experience.

## Introduction

Parkinson's disease (PD) is a chronic, progressive neurodegenerative disorder that diminishes motor ability and quality of life in over 1.5 million people in the USA and 7 to 10 million people worldwide (1). In addition to difficulties with straight walking, turning and negotiating both predictable and unpredictable environments associated with community accessibility are significant problems for people with PD and greatly impact participation in societal roles (2–4). Importantly, gait disorders respond poorly to dopaminergic replacement therapy (5–7). Physical activity has consistently been identified as an effective, non-pharmacological intervention for improving motor performance in PD (8). As a result, clinicians and researchers are actively searching for means to increase lifelong participation in physical activity for people with PD. Virtual reality (VR)-based mobility training is a promising tool to provide an enjoyable, engaging, and enriched setting for forms of physical therapy capable of improving functional mobility in older adults, people post-stroke, and individuals with PD (9–11).

Two specific aspects of VR make it an ideal platform within which people with PD can practice complex gait skills. First, the emergence of consumer-level, “roomscale” VR systems has widened the feasibility of using VR during the performance of a broad range of locomotor activities. Room-scale VR applications allow the user to physically move around a given space and practice both straight walking and turning. Secondly, virtual environments allow for the natural and seamless integration of motor learning practice variables known to optimize long-term retention of learned skills. Environmental context (12, 13), motivation (14), and external attentional focus (15) are three specific practice variables that have recently emerged as being particularly influential in PD and highly amenable to control within virtual environments.

Despite the aforementioned benefits of incorporating VR in skill training for people with PD, previous studies have demonstrated inconsistent results when VR-based interventions are compared to conventional approaches for improving gait and balance (11, 16, 16–21). Many studies have used non-immersive off-the-shelf (17, 20, 22, 23) or custom games (16) that focus primarily on balance training as their VR-based intervention. Other studies have used bespoke games created for mobility training. Although these games allow participants to negotiate virtual obstacles and select different virtual paths, participants are unable to practice turning because they are constrained to walk on a treadmill (18, 19). To date, there have been no VR-based mobility training applications that allow users to practice skills such as turning and obstacle negotiation with simultaneous problem-solving while walking over-ground. A fundamental assumption of the use of VR for motor skill training is that the skills learned in the virtual environment will transfer to the real world. However, the degree of transfer is known to depend on the similarity between the training environment and the real-world context in which the learned skill is to be performed (24–26). By incorporating skills such as turning and obstacle negotiation in combination with problem-solving in a fully immersive, over-ground training system, it may be possible to enhance the transfer of locomotor skills for people with PD beyond what has been observed in non-immersive systems that focus on balance or treadmill-based walking.

Evaluations of VR-based interventions typically focus on determining efficacy relative to the current standard of care. Still, even efficacious VR interventions may have limited clinical translation because the needs of the stakeholders have not been considered throughout the design and evaluation process. There are many known barriers and facilitators to clinical translation of VR-based training interventions including “the degree of match between the system and the client's goals/needs, the ability to grade the degree of training, transfer of training to real life, (…) knowledge about how to operate and to apply the technology clinically, therapist self-efficacy and perceived ease of use, perceived utility (…) technical and treatment space issues, access, time to learn/practice and use the technology, support for setup/takedown and administering treatment, client and therapist motivation (27, 28).” Each of these barriers and facilitators necessitates a user-centered design approach with a wide range of stakeholders who need to be involved during formative research of VR interventions and not just the recipients of care (the patients).

In this paper, we describe the design and development of a VR-based mobility training application for people with PD. We first describe our iterative, user-centered needs assessment, where we consulted with people with PD and physical therapists to determine the design specifications for our system. We then describe the development and usability assessment of our mobility training application, where people with PD completed a set of three progressive, 30-min sessions under the direction of their current physical therapist. We paired our PD participants with their therapist because they were already working together on common goals, and their therapist would be best able to match the parameters of the training application to their client's needs. Together, the outcomes of this process provide solutions to the challenges mentioned above that have limited both the real-world transfer of skills learned in VR and the integration of VR-based interventions into clinical practice. In trying to address the barriers and facilitators of clinical translation, this study went beyond the evaluation of system usability to measure “entertainment efficacy,” which, for VR-based health games, includes intrinsic motivation and presence.

## Methods

### Participants

We recruited 17 total participants for the study (Table 1), including nine people with PD (64 ± 12 years) and eight physical therapists (PT, 36 ± 10 years). Physical therapists were recruited by contacting known neurological clinical specialists who worked with people with PD in the greater Los Angeles area. Physical therapist participants selected an eligible person with PD among their patients. We selected our sample size to be larger than the accepted sample size of five users per software iteration for usability testing (8, 29, 30). Moreover, our statistical analyses focused on within-subject differences, consistent with the repeated-measures design of our assessments.

**Table 1:** Participant characteristics.

**Group**	**Age range**	**Years since diagnosis**	**MDS-UPDRS (III)**	**H&Y**	**Mini-BEST**
PT	35–39	NA	NA	NA	NA
PT	30–34	NA	NA	NA	NA
PT	25–29	NA	NA	NA	NA
PT	40–44	NA	NA	NA	NA
PT	30–34	NA	NA	NA	NA
PT	55–59	NA	NA	NA	NA
PT	25–29	NA	NA	NA	NA
PT	35–39	NA	NA	NA	NA
PD	70–74	11	7	2	18
PD	45–49	2	24	1	27
PD	65–69	9	29	1	28
PD	45–49	12	28	3	23
PD	70–74	6	47	3	16
PD	60–64	2	15	1	26
PD	65–69	19	26	3	17
PD	75–79	1	29	2	23
PD	65–69	11	26	2	22

*H & Y, hoehn and yahr stage; MDS-UPDRS: Mini-BEST: Mini Balance Evaluation Systems Test; MDS-UPDRS, movement disorders society–unified Parkinson's disease rating scale; PD, participants with Parkinson's disease; PT, physical therapists*.

Potential participants with PD were eligible for our study if they were diagnosed with idiopathic PD with no motor fluctuations, had Hoehn and Yahr scores between 1 and 3 (mild to moderate PD), were greater than or equal to 18 years of age, and were walking independently. Participants were also required to be stable with their pharmacological treatment without differing in Hoehn and Yahr staging between medication-off and medication-on conditions. Potential participants were excluded if they showed side effects such as uncontrolled, involuntary movements (dyskinesia), if they had musculoskeletal injuries, or if they had pain that limited their movement. Participants were always tested while they were on their routine PD medication. Physical therapists were eligible to participate in our study if they had expertise in the treatment of people with PD and were currently treating an eligible participant with PD.

Participants were informed that they would place a VR headset onto their head, hold a controller in each hand, and have a set of sensors attached to their waist and on top of each foot. During the session, participants viewed the virtual environment through the headset, heard different sounds when completing tasks, and felt slight vibrations from the hand controllers. They were told that they would be asked to perform tasks within the virtual environment, such as reaching out to virtually grasp objects, stepping over or around obstacles, and turning. Lastly, participants were informed that they would be asked to complete a set of computer-based questionnaires regarding their experience after the end of the VR session. The Institutional Review Board at the University of Southern California approved the study protocol, and all participants provided informed consent before participating. All aspects of the study conformed to the principles described in the Declaration of Helsinki.

### Application Design

The software used in this study was developed by the authors through a user-centered, participatory design process that incorporated feedback from people with PD and physical therapists throughout the development phase. The *Wordplay* game used as the intervention in this study was the result of a multi-year process of formative research. This process helped identify barriers to community mobility in people with PD, such as environments with people or things that move in irregular patterns (e.g., crowds, road crossings), externally imposed time pressure, performing simultaneous cognitive tasks, and increased anxiety (17). In addition, the fact that people with PD have difficulty turning (17, 18) and are more likely to fall from tripping over obstacles than age-matched controls (19) informed the design brief describing the types of tasks that were to be elicited from players in the virtual environment. We also identified and subsequently incorporated into the design key principles known to modulate the efficacy of motor skill learning (14). These included practicing skills in multiple environmental contexts, enhancing motivation for practice, and focusing attention on the outcomes of one's movements.

The specifications in the design brief aimed to (1) address specific functional limitations of people with PD, (2) integrate gameplay features that provide a low barrier to use, (3) motivate the patient, (4) stimulate a desire for replaying, and (5) incorporate principles of motor skill learning. In the second stage, we selected, created, and combined key hardware and software assets to produce a unique system with a corresponding set of mobility training tasks. This system allows people with PD to practice tasks such as walking, reaching, turning, obstacle negotiation, and problem-solving in a fully immersive, 3D virtual environment.

The objective of *Wordplay VR* was for users to complete a puzzle that consisted of a word with missing letters located at eye level in the virtual environment. The player had to determine which letters were necessary to complete the puzzle, collect the necessary virtual letters as they floated in 3D space, and then place the letters in the appropriate location. This training application was specifically designed to encourage people with PD to practice walking, reaching, turning, obstacle negotiation, and dual-tasking in a fully immersive, 3D virtual environment. The level of challenge and the required speed of movement were customized on an individual basis by varying word difficulty, the number of missing letters, the time allotted to complete the task, the spatial distribution of solution letters, whether the solution letters disappeared and reappeared, and whether participants had to negotiate virtual obstacles simultaneously.

### Hardware

**Figure 1:**
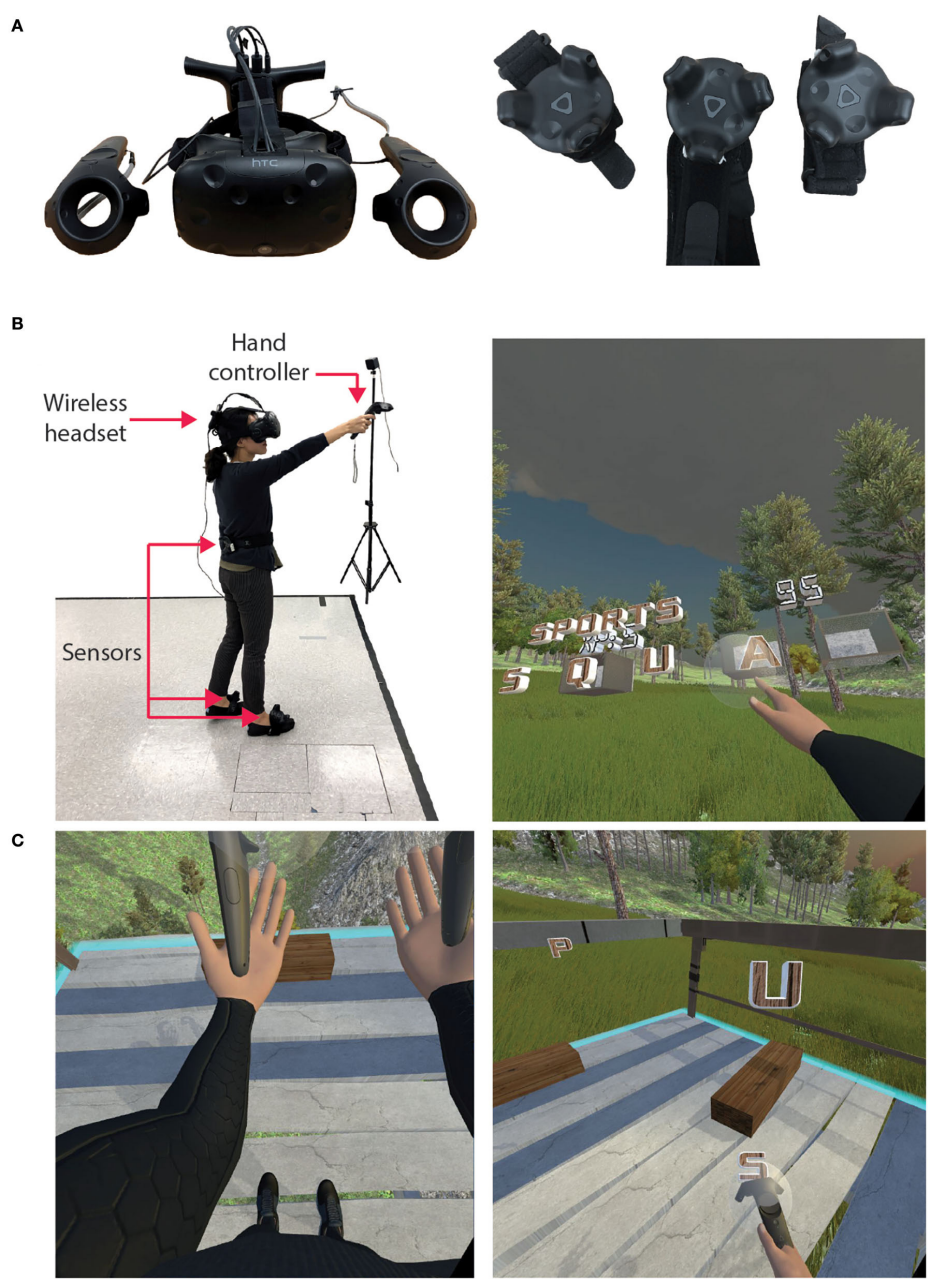
VR hardware and in-game views. **(A)** HTC Vive headset, hand controllers, and sensors. **(B)** A third-person outside view and first-person inside view of Wordplay VR. An individual donned the wireless headset and held a controller in each hand. HTC Vive trackers were worn on the feet and the lower back. Participants' movement was tracked in real time and presented in VR using an avatar. **(C)** In-game views of the avatar, virtual obstacles, and solution letters during gameplay.

We used the HTC Vive (HTC Corporation, USA) to allow participants to interact with the Wordplay VR experience (Figure 1A). The HTC Vive is a head-mounted display (HMD) with a 100° field of view, a resolution of 1,080 × 1,200 pixels per eye, and a frame rate of 90 Hz. The HTC Vive was equipped with a wireless adapter that allowed participants to walk in the virtual environment without being tethered to a computer. Additionally, the system used two HTC controllers, two Vive trackers on the feet, and one Vive tracker on the trunk to control an avatar (Figures 1B,C) and interact with the virtual environment. The HMD and trackers were tracked by two lighthouse cameras that were placed in opposite corners of the 3 × 3 m play area. The application was programmed in Unity, and the avatar was rendered and controlled via the IKINEMA Orion plugin.

### Protocol

All data collection sessions were completed at the University of Southern California Locomotor Control Lab in Los Angeles, California. Participants completed each of the following self-assessments by completing questionnaires through the web-based Research Electronic Data Capture (REDCap) application.

**Figure 2:**
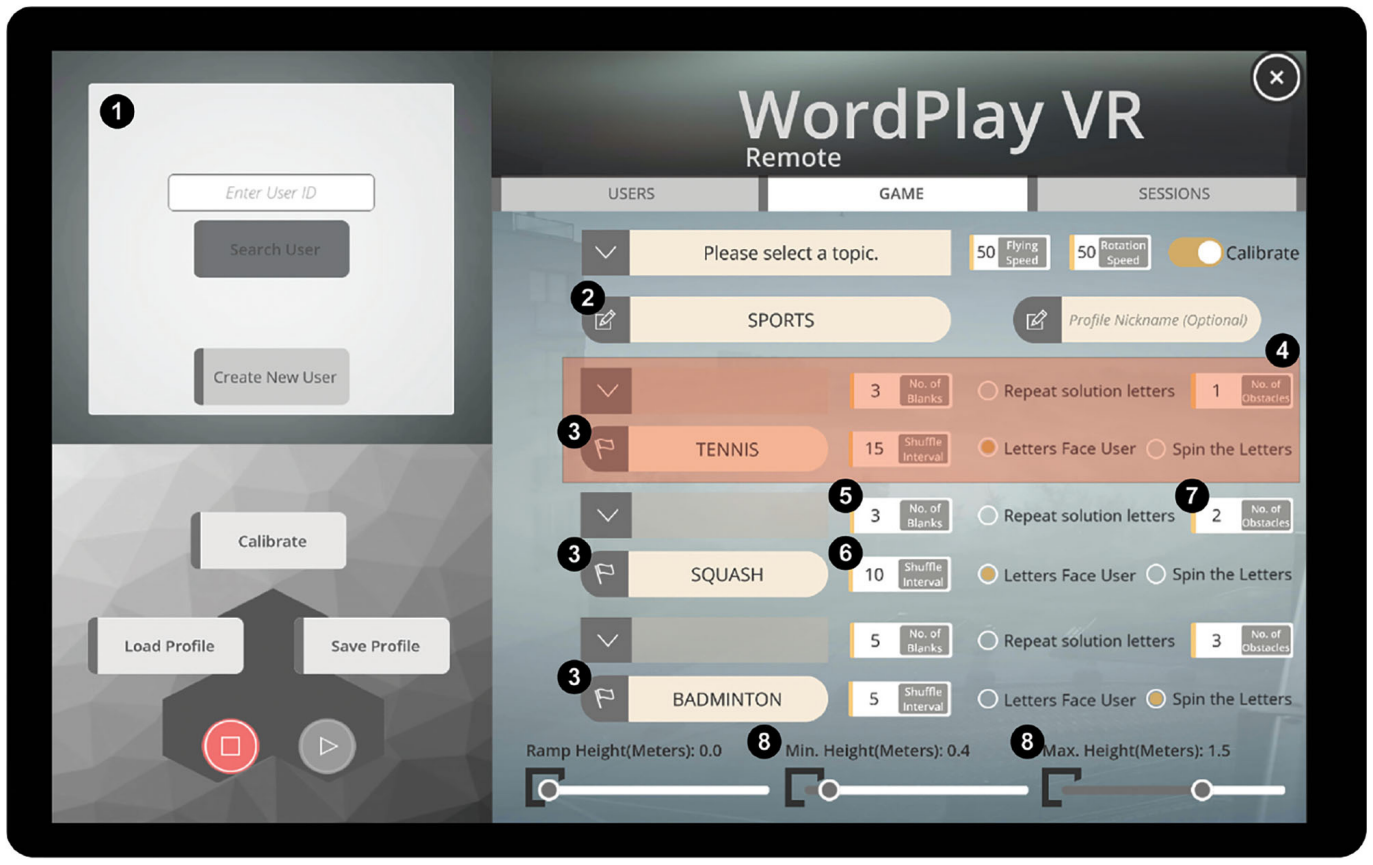
Tablet-based user interface. (1) User profile menu, (2) category for the current session, (3) puzzle words for each of three rounds, (4) settings for a single round, (5) number of missing letters in the puzzle, (6) time interval between letter movements, (7) number of virtual obstacles, and (8) range of heights at which the solution letters could be placed.

Patient–therapist pairs completed three training sessions using our system over 1 week (Table 2). During the first session, we assessed the therapists' baseline levels of symptoms of simulator sickness (31), and then they completed 10 min of gameplay. After completing the 10-min session, they completed a post-test assessment of simulator sickness and the System Usability Scale (SUS) (32) to quantify potential adverse effects and perceived usability. They also completed the Interest and Value subscales of the Intrinsic Motivation Inventory (IMI) (33) from the perspective of a player within the game. Next, they completed a tutorial to learn how to navigate the tablet-based user interface (Figure 2) and select training parameters for their patients.

**Table 2:** Testing protocol.

	**Day 1**			**Day 2**			**Day 3**	
	**Pre-test**	**Post-tutorial**	**Post-play**	**Pre-play**	**Post-play**	**Pre-play**	**Post-play**
Unified Parkinson's disease rating scale	PD						
mini-BESTest	PD						
**Safety**							
Simulator sickness questionnaire	PD, PT	PT	PD	PD	PD	PD	PD
**User experience**							
System usability scale (play)		PT	PD		PD		PD
System usability scale (control)			PT		PT		PT
Intrinsic motivation inventory (play)		PT	PD		PD		PD
Intrinsic motivation inventory (control)			PT		PT		PT
ITC sense of presence inventory		PT	PD		PD		PD

While the therapist completed their baseline assessments, we assessed the level of motor dysfunction in our participants with PD using the Movement Disorder Society-Unified Parkinson Disease Rating Scale (MDS-UPDRS) part III (34). Then, we performed baseline measures of their dynamic gait and balance using the Mini-Balance Evaluation Systems Test (Mini-BESTest) (35). After the clinical exams were complete, we performed a baseline assessment of simulator sickness in our participants with PD to capture any baseline symptoms of discomfort associated with the disease. They then completed 15 min of training in the virtual environment after their therapist selected the appropriate training parameters through the tablet-based interface.

After the training session, participants with PD completed a Simulator Sickness Questionnaire, the ITC Sense of Presence Inventory (36), the IMI, and the SUS (32) to evaluate adverse effects, sense of presence, levels of motivation, and overall system usability, respectively. Our PT participants also completed the IMI and the SUS from the perspective of their role in controlling the training session. During the second and third visits, patients completed 20 min of training, and then both the patients and PT participants completed the same set of questionnaires as the first session. We also recorded the time required for the therapists to set up the system so that we could evaluate how the use of the system in a therapeutic setting might impact the time available for therapy.

### Outcome Measures

The Independent Television Commission Sense of Presence Inventory (ITC-SOPI) (36) is a 44-item survey used to assess three domains of “being there”: physical space (spatial presence), engagement, and ecological validity/naturalness. The Spatial Presence subscale evaluates the extent to which the player felt as if they were actually in the virtual space. The Engagement subscale evaluates how psychologically involved the player was in the game and how much they enjoyed it. The Ecological Validity subscale assesses whether the user perceived the virtual environment to be like real life. Each response was provided on a five-point Likert scale (1 = strongly disagree; 5 = strongly agree). We computed the total score for each subscale by calculating the mean value of all questions for the respective subscale.

We assessed participants' subjective experience with the virtual environment using the interest/enjoyment and value/usefulness subscales of the IMI (33). Each subscale was scored on a seven-point Likert scale. For data analysis, negative items were reverse-scored, and subscale scores were calculated by averaging across all items on each subscale. A higher score indicates a greater contribution from the concept described in the subscale name. We customized three items on the value/usefulness subscale to make them specific to our training environment. These items were customized as follows: “I think that doing this activity is useful for [increasing mobility],” “I think this is important to do because it can [increase mobility],” and “I think doing this activity could help me [increase mobility].” An overall IMI score was generated by calculating the mean score of all subscales. Based on previous experience with this instrument and prototypes of interactive entertainment, we set benchmarks of 75% of participants rating the game higher than the scale midpoint for the interest/enjoyment subscale and 50% of participants rating the game higher than the midpoint for the value/usefulness subscale.

The SUS was used to measure overall system usability (32). The SUS is a 10-item questionnaire with five response options ranging from “Strongly agree” to “Strongly disagree.” Based on data from ~500 studies using the SUS, we used a score of 68 to denote the threshold for above-average overall usability (37, 38).

We measured the time required for therapists to help their patients don the VR headset and trackers, specify the gameplay parameters in the user interface, and begin gameplay.

Levels of symptoms associated with simulator sickness were measured using the Simulator Sickness Questionnaire (SSQ) (31). The SSQ includes 16 questions related to symptoms of simulator sickness, and we used the questionnaire to detect changes in symptoms of nausea, oculomotor discomfort, or disorientation due to exposure to the virtual environment. Participants answered each of the 16 questions based on the severity of symptoms they experienced at the moment using a four-point scale from “none” to “severe” (0–3). A cutoff score of 20 was used to determine if participants experienced significant simulator sickness after exposure (39).

### Statistical Analysis

We performed a Wilcoxon signed-rank test to determine if our PT participants increased their SSQ scores after exposure to the virtual environment on Day 1. Similarly, we performed Friedman's test to determine if there was a significant increase in SSQ scores in our patients with PD after exposure to the virtual environment or across days. We also performed a non-parametric Friedman's test to determine if there were statistically significant changes in measures of simulator sickness from baseline to after the gameplay period. We performed a non-parametric Friedman's test on the measures of setup time to determine if our PTs improved their proficiency using the system following repeated sessions. For the ITC-SOPI, IMI, and SUS, we report median values and interquartile ranges for each day and each user group. We do not perform formal statistical analyses of these metrics as we were primarily interested in using the scores for a qualitative evaluation of our system.

## Results

### Simulator Sickness and Safety

**Figure 3:**
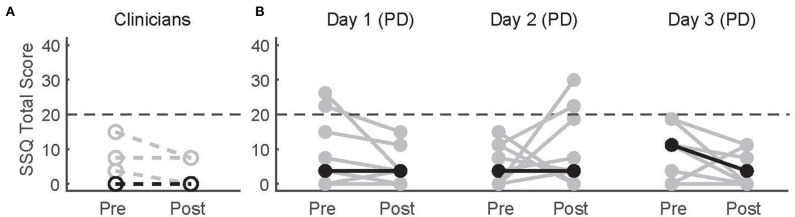
Pre-test and post-test SSQ scores for **(A)** physical therapists and **(B)** patients with PD across three sessions. Gray data points correspond to different participants. Black points correspond to median scores across participants for each time point. Lines connect the pre- and post-scores for each participant. The horizontal dashed line indicates the threshold for significant adverse symptoms of simulator sickness.

Simulator-related sickness symptoms measured by the SSQ overall did not change after WordplayVR sessions or across days in patients with PD (Figure 3). There was no significant difference in the SSQ total scores between pre- and post-WordplayVR on Day 1 in PTs (*p* = 0.5). Moreover, there was no significant effect of time point (pre vs post, *p* = 0.62) or day (*p* = 0.86), nor was there a significant interaction between time point and day (*p* = 0.16) on the SSQ scores in patients with PD. Only two patients with PD increased their SSQ scores after playing WordplayVR above the threshold of 20, which is the benchmark score for having symptoms of simulator sickness, and this occurred on Day 2. These increases resulted from an increase in nausea-related symptoms. We did not observe any falls or other adverse effects in any of our study participants.

### Sense of Presence

**Figure 4:**
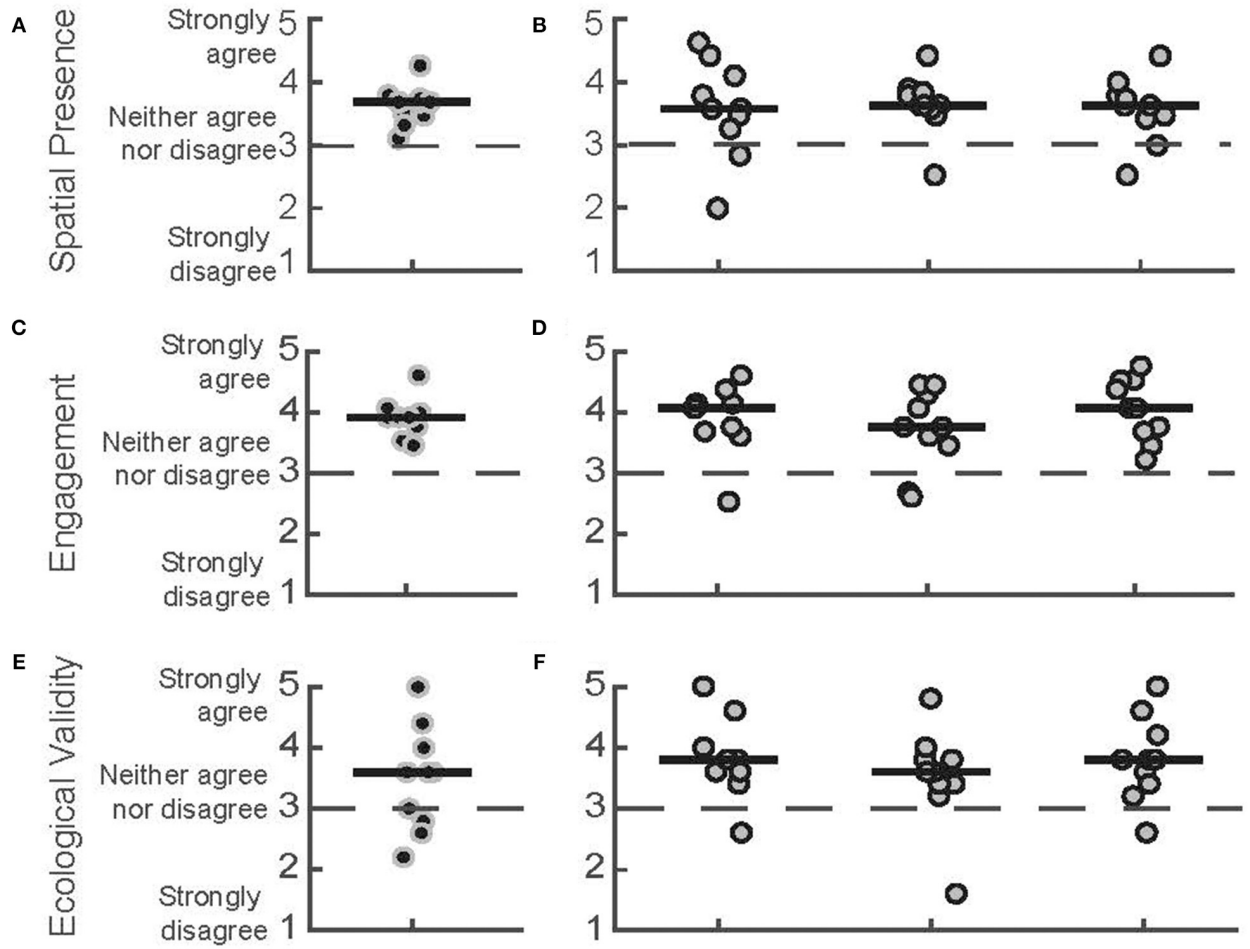
ITC-SOPI subscale scores. Spatial presence scores for **(A)** PTs during the first day of play and **(B)** patients with PD for 3 days of play, respectively. Engagement scores for **(C)** PTs and **(D)** patients with PD. Ecological Validity scores for **(E)** PTs and **(F)** patients with PD. Each data point corresponds to a different participant. Black points correspond to PTs, and gray data points correspond to participants with PD. Median scores for each day are indicated by solid horizontal lines. The score corresponding to a neutral response is indicated by the horizontal dashed line.

We found overall agreement with the statements relating to Spatial Presence, Engagement, and Ecological Validity, as seen in the ITC-SOPI responses from both the PTs and the patients with PD (Figure 4). Participants from both groups generally agreed with the statements comprising the Spatial Presence subscale, which was indicated by group medians >3 [PTs = 3.68 (IQR = 3.47–3.74), PD day 1 = 3.58 (3.26–4.10), day 2 = 3.63 (3.58–3.84), day 3 = 3.63 (3.42–3.79)]. The responses for the Engagement subscale followed a similar trend, with both groups generally agreeing with the related statements [PTs = 3.92 (3.77–4), PD day 1 = 4.08 (3.69–4.15), day 2 = 3.77 (3.46–4.31), day 3 = 4.08 (3.69–4.54)]. Participants from both groups also generally agreed with the statements related to the Ecological Validity subscale as the median scores were all >3 [PTs = 3.6 (2.8–4), PD day 1 = 3.8 (3.6–4), day 2 = 3.6 (3.4–3.8), day 3 = 3.8 (3.4–4.2)]. Median scores across all subscales for the PD group were similar across the three sessions, suggesting that neither their experience of the virtual space, their engagement, or their perceptions of ecological validity changed with repeated exposure.

### Intrinsic Motivation While Playing Wordplay VR

**Figure 5:**
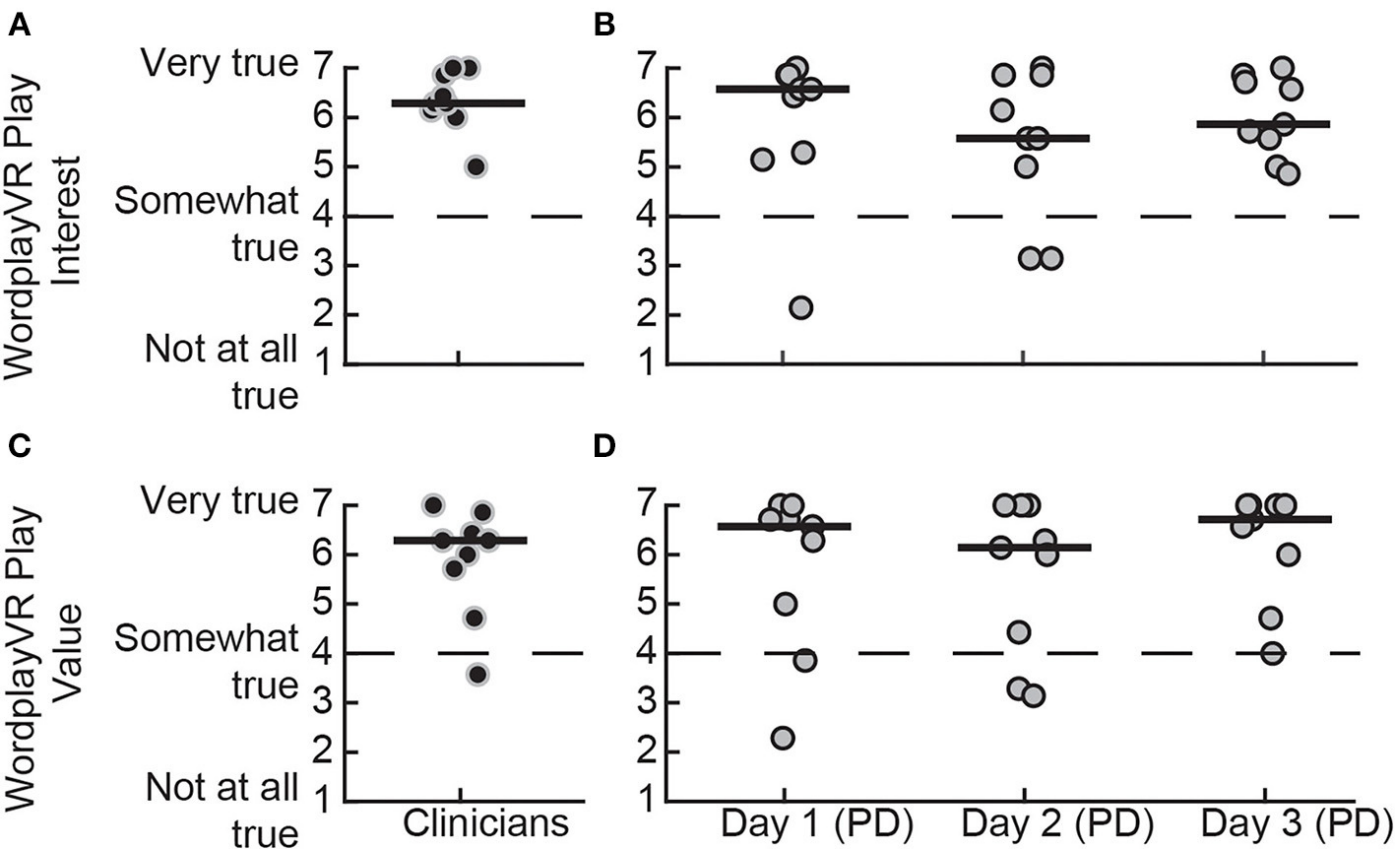
IMI subscale scores for playing WordplayVR by PTs and patients with PD. Interest subscale score for **(A)** PTs during the first day of play and **(B)** patients with PD for the 3 days, respectively. Value subscale score for **(C)** PTs and **(D)** patients with PD. Black data points correspond to PTs, and gray data points correspond to participants with PD. Median scores for each day are indicated by solid horizontal lines. The score corresponding to a neutral response is indicated by the horizontal dashed line.

We used the IMI to assess the participants' subjective sense of interest and value of the gameplay experience (Figure 5). Both PTs and people with PD responded positively to their experience during the play sessions, with the median scores for both groups being above the neutral point of four. This was observed for both the Interest [PTs = 6.29 (6.11–6.89), PD day 1 = 6.57 (5.25–6.86), PD day 2 = 5.57 (4.54–6.86), PD day 3 = 5.86 (5.43–6.75)] and Value [PTs = 6.29 (5.46–6.54), PD day 1 = 6.57 (4.71–6.79), PD day 2 = 6.14 (4.14–7), PD day 3 = 6.71 (5.68–7)] subscales. Additionally, the scores of the PD group for both scales remained stable over the 3 days, suggesting that intrinsic motivation remained high across all play sessions.

**Figure 6:**
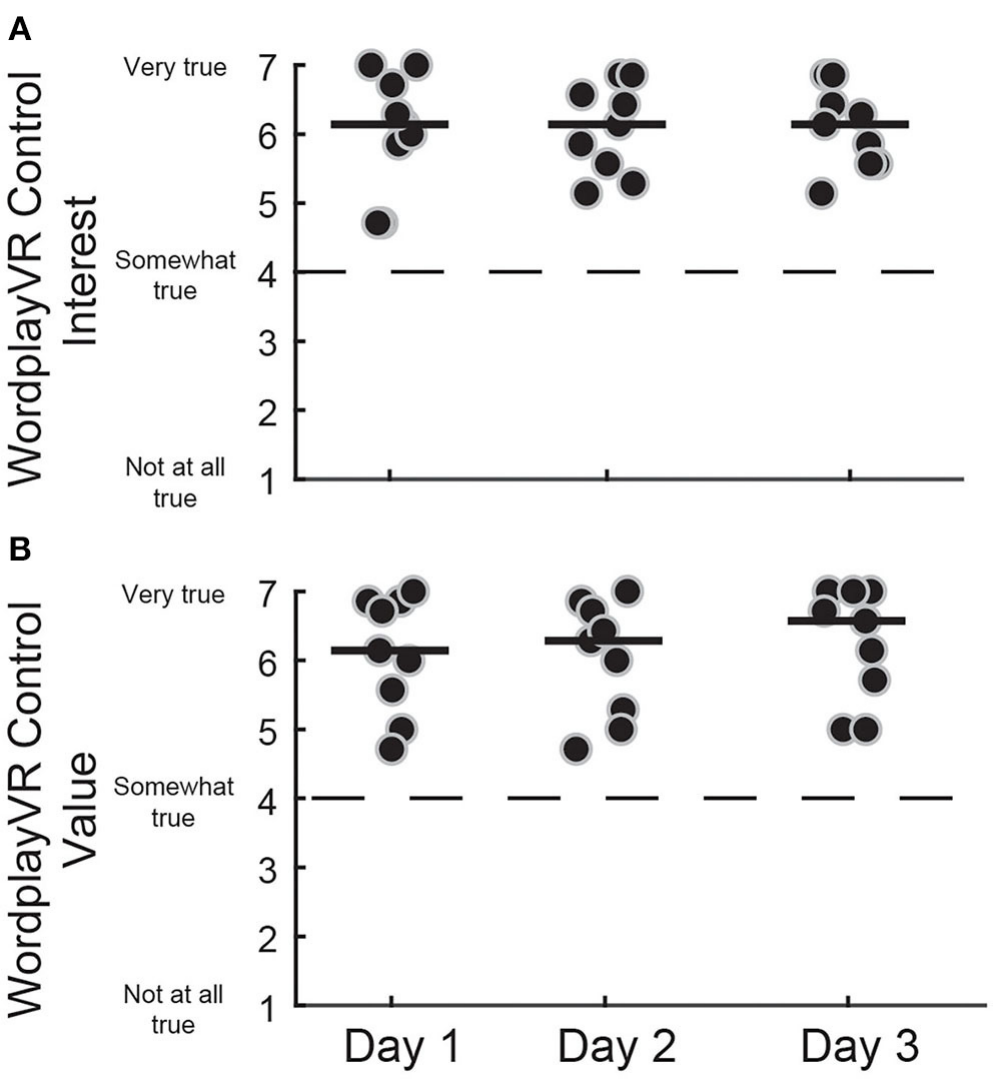
IMI subscale scores for controlling WordplayVR software by PTs. **(A)** Interest subscale for three consecutive days of WordplayVR experience. **(B)** Value subscale. Black data points correspond to individual PTs. Median scores for each day are indicated by solid horizontal lines. The score corresponding to a neutral response is indicated by the horizontal dashed line.

We also administered the IMI to the PTs at the end of each day to assess their intrinsic motivation from the perspective of the director of the training session (Figure 6). All the PTs provided high scores, all above the neutral point of four, on all 3 days. This was observed for both the Interest [Day 1 = 6.14 (5.57–6.79), Day 2 = 6.14 (5.50–6.64), Day 3 = 6.14 (5.57–6.54)] and the Value [Day 1 = 6.14 (5.43–6.86), Day 2 = 6.29 (5.21–6.75), Day 3 = 6.57 (5.54–7)] subscales. The responses were consistent across the 3 days, which indicates that the PTs did not lose interest in directing the gameplay session and did not perceive the value of the game to diminish with repeated sessions.

### System Usability

**Figure 7:**
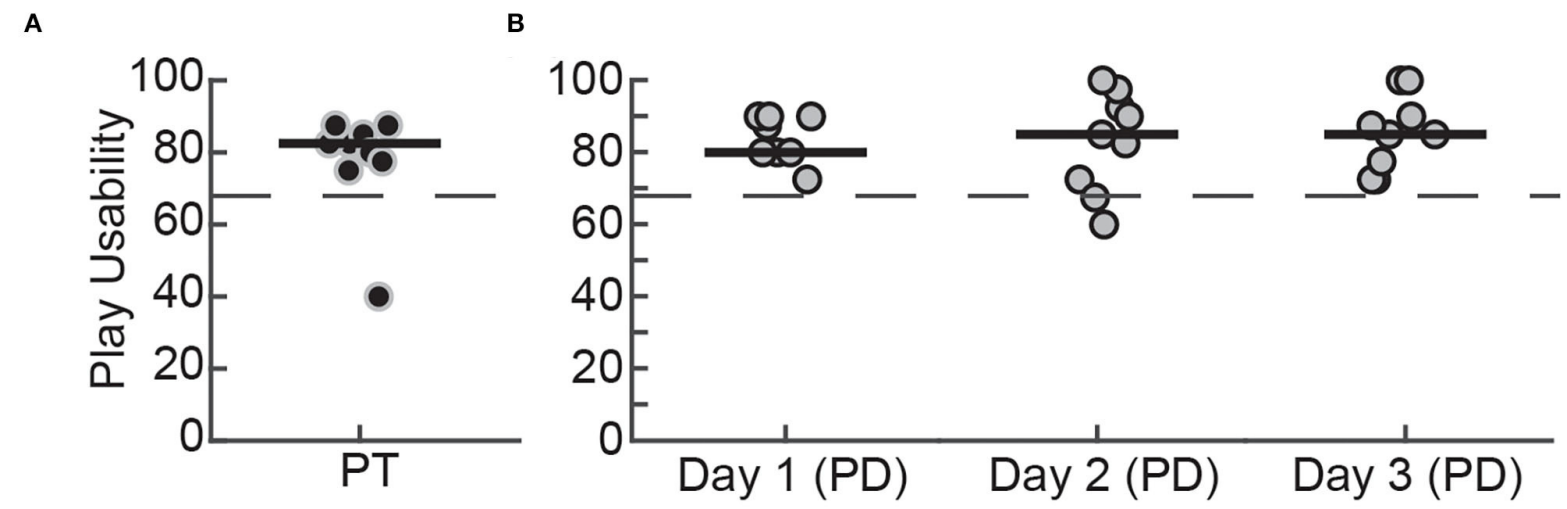
SUS scores for playing WordplayVR by **(A)** PTs on the first day of play and **(B)** patients with PD for 3 days. Black data points correspond to PTs, and gray data points correspond to participants with PD. Median scores for each day are indicated by solid horizontal lines. The horizontal dashed line indicates the threshold of 68, which constitutes an average level of overall usability.

**Figure 8:**
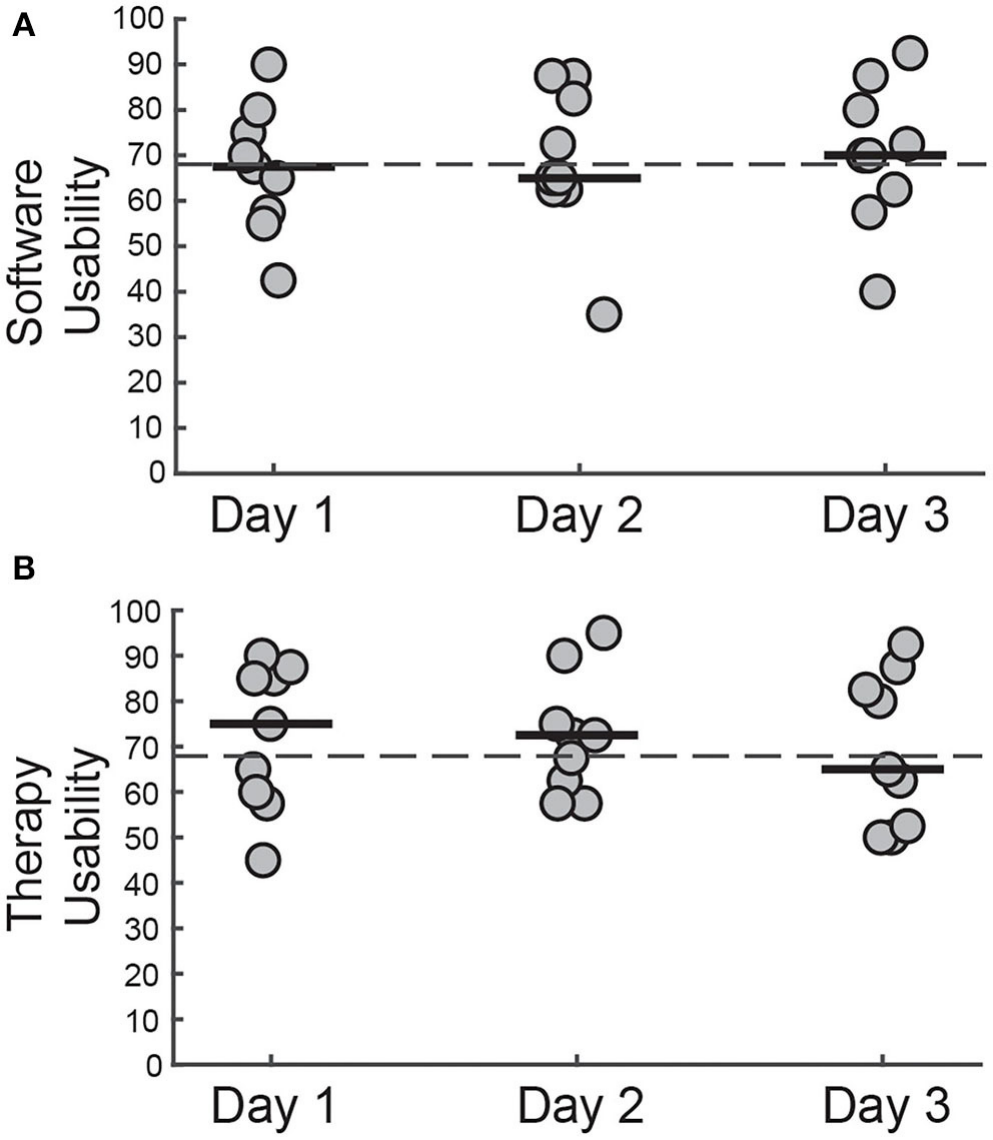
SUS scores for **(A)** controlling WordplayVR software and **(B)** using WordplayVR in physical therapy practice by PT for 3 days. Each data point corresponds to a different PT. Median scores for each day are indicated by solid horizontal lines. The horizontal dashed line indicates the threshold of 68, which constitutes an average level of overall usability.

We evaluated the usability of WordplayVR by measuring responses to the SUS from three user perspectives. These perspectives included (1) usability within the game as a player (Figure 7), (2) usability of the user interface by PTs (Figure 8A), and (3) usability for physical therapy practice (Figure 8B). From the perspective of playing the game, participants rated the system's usability highly throughout the play sessions [PTs = 82.5 (76.87–85.62), PD (Day 1) = 80 (80–90), PD (Day 2) = 85 (71.25–93.75), PD (Day 3) = 85 (76.25–92.50)].

Concerning the user interface, the median scores provided by the PTs across the 3 days were close to 68, which is generally considered to be an average overall usability score [Day 1 = 67.5 (56.87–76.25), Day 2 = 65 (62.50–83.75), Day 3 = 70 (61.25–81.87)]. The therapy usability scores also fluctuated around 68 for each of the 3 days [Day 1 = 75 (59.37–85.62), Day 2 = 72.5 (61.25–78.75), Day 3 = 65 (51.87–83.75)]. Individual scores indicated that all PTs perceived Wordplay VR as being acceptable or marginally acceptable for use in a therapy setting (40).

### Setup Time

**Figure 9:**
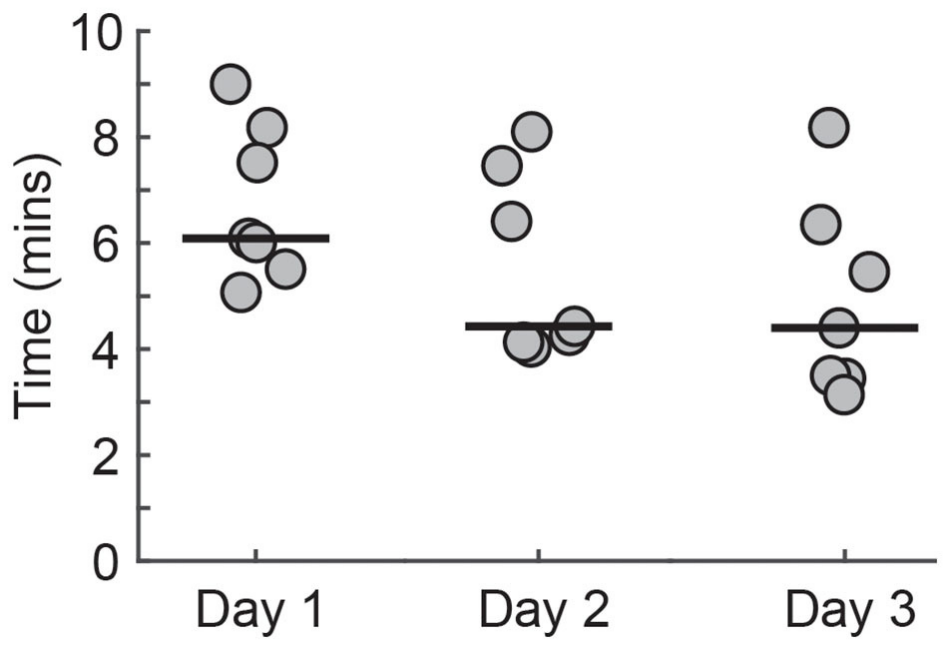
The time required for the PTs to set up Wordplay. Each data point corresponds to a different PT. Median scores for each day are indicated by solid horizontal lines.

All PTs reduced the time required to set up the system across the three sessions (Figure 9). There was a trend toward a reduction in setup time across days [*F*(2, 12) = 3.87, *p* = 0.05]. The time taken for setup decreased by over 25% from the first to the third session [Day 1 = 6.09 min (5.63–8.01), Day 2 = 4.43 min (4.16–7.20), Day 3 = 4.40 min (3.46–6.13)].

## Discussion

We developed and evaluated a custom, VR-based mobility training application for people with PD. Our application provided a game-based environment in which people with PD could practice skills such as turning, obstacle avoidance, and navigating unpredictable environments while walking over-ground. The gameplay parameters were easily modifiable by the therapist, and this allowed them to create personalized levels of challenge for their patients. Our primary objective was to evaluate the usability of our system from the perspective of our two primary populations of end users: people with PD and their physical therapists. We found that both groups of participants provided high ratings on the interest and value subscales of the IMI and provided assessments of the system's usability that were equal to or above the average score for many types of products across a wide range of development stages (38). We also found no evidence of adverse effects following exposure to the virtual environment, and this suggests that our application is unlikely to produce adverse effects in people with similar characteristics to our study sample. Our design and evaluation framework addressed several previously acknowledged barriers and facilitators to clinical translations of VR-based training applications. As a result, this framework could also be applied in future studies to integrate potential end users in the development pipeline and improve the likelihood that newly developed VR interventions will be clinically viable.

### ITC-SOPI

We administered the ITC-SOPI to our participants for an evaluation of their experience with the game. This scale is used to evaluate the overall sense of presence, which is a subjective measure of the extent to which users feel that they are inside the virtual space even though they are physically elsewhere (41). We assessed three subscales of this questionnaire—Sense of Physical Space (Spatial Presence), Engagement, and Ecological Validity. We found general agreement with the statements relating to Spatial Presence, Engagement, and Ecological Validity, as seen in the responses by the physical therapists and the people with PD. The agreement was indicated by scores greater than the scale's neutral midpoint of three. The scores of both groups tended to be similar, and no large differences between them were observed. Additionally, the responses of the PD group were similar across all 3 days, suggesting that repeated exposure did not diminish their experience while playing the game. A recent study that compared users' sense of presence while playing a game with a head-mounted VR device vs. a traditional computer screen found greater presence in the VR group, with the group mean presence score being 3.44 (42). This is comparable to our group median score for presence.

### Interest and Value of Wordplay VR

We evaluated the Interest and Value subscales of the IMI to assess participants' motivation levels while playing the game and the therapists' motivation levels while controlling the game sessions. We found high levels of motivation for both groups while playing the game, indicated by their responses on both subscales, which were greater than the midpoint of four. Additionally, the PTs also indicated high levels of motivation while controlling the game sessions. The responses of both groups were consistent across the 3 days, suggesting no reduction in motivation. Lloréns et al. (43) assessed a VR-based telerehabilitation system for people post-stroke and found mean Interest and Value scores to be 6.16 and 6.12, respectively. A similar study in people post-stroke evaluated VR-based intervention reported scores of 5.46 and 5.66, respectively (44). Both of these sets of scores were comparable to our study as we observed Interest and Value scores on Day 3 for people with PD of 5.86 and 6.71, respectively. One of the unique features of our study is that we also evaluated interest and value from the point of view of the therapists who controlled the game. This has not been previously reported, but it is critical as therapists must perceive that the system has value for clinical practice for the system to be used in a therapeutic setting.

### Usability of Wordplay VR From the Perspective of Patient and Therapist

Despite considerable interest in VR-based interventions, VR applications for physical rehabilitation are often not tested for usability. Usability evaluation should consider not only ease of use, but also utility for therapeutic purposes (40). Therefore, we investigated system usability in the context of playing Wordplay VR as well as usability from the perspective of how physical therapists would use the application in a therapeutic setting. Both people with PD and physical therapists rated our application as having acceptable levels of usability while playing Wordplay VR, and these perceptions were consistent across multiple days. These findings are consistent with a recent systematic review on usability issues of VR applications, which found that older adults perceived training applications in immersive VR acceptable or marginally acceptable (40). Moreover, physical therapists perceived that applying Wordplay VR in a therapeutic setting to be at least marginally acceptable, and the result held across multiple days. Novel rehabilitation techniques do not necessarily have good usability by default. For example, a previous study testing the acceptability of a biofeedback device by physical therapists found that several testers rated the device to have poor acceptability (45). The poor acceptability was primarily due to the excessive complexity of the device. During the development of our application, we intentionally designed the interface to be easy to use, and this was reflected in the therapists' usability assessment. However, the lab-based setting in which we assessed usability differs markedly from conventional practice environments.

By addressing the previously described issues that have led to minimal carryover between VR research and real life, we have developed a system that provides several potential solutions to achieving a seamless transition between lab-based VR systems and the use of these systems in clinical practice. For one, the design of our system is based almost entirely on input from the stakeholders, both physical therapists and individuals with PD, creating a match between the system and the client's goals/needs. Secondly, our system is scalable through customizable levels of challenge, thus providing the ability to tailor and grade the degree of training for each client regardless of disease severity. Likewise, motivation is enhanced by the ability of the system to capture an individual's progress in managing increasingly greater levels of challenge over practice. Third, our effort was not only the development of the VR system but in training the physical therapists in how to operate and to apply the technology. Designing intuitive user interfaces and training clinicians to use interactive technologies for rehabilitation has long been recognized as an ongoing challenge in this field (46, 47). Over the three intervention sessions, therapists were given the time to learn/practice and use the technology, including setup and takedown. Therapists were motivated by their improved skill and efficiency, which they developed through practice setting up the system. This is promising for any clinical setting where physical therapists may be hesitant to implement technologies in practice due to excessive setup time costing precious patient care time.

## Limitations

As an early-stage, development, and proof-of-concept study, there are several features of the study's design that limit the extent to which our results can be used to inform the use of VR-based interventions for improving mobility in people with PD. First, our system was evaluated by a small set of clinicians and people with PD, and as a result, it remains to be seen if our results will generalize to a larger, more diverse sample. For example, since we did not include a cognitive assessment of our PD participants, it remains to be seen if the perspectives provided by our participants would be shared by individuals with cognitive impairment. This is important because mild cognitive impairment may be present in ~25% of people with PD (48) and this can often progress into dementia (49). Since our training platform requires problem-solving, working memory, and visual search, it is possible that individuals with cognitive impairment could find the task to be overly challenging. However, this possibility could be mitigated in part through careful specification of the training variables by the physical therapist. Second, the assessment tools that we used, including the IMI, SUS, and ITC-SOPI, have not been validated in people with PD. As a result, the interpretation of the scores on these assessments relative to reported cutoff values could be inaccurate if our participants exhibited any systematic biases or inconsistencies in how they responded to these questionnaires. However, the observed day-to-day consistency of these outcome measures suggests that, at the very least, the evaluations that people with PD provided are reliable. Lastly, our training dose was not designed to be large enough to evaluate potential benefits of training with our system on gait and balance.

## Future Directions

Despite the promising usability results we observed in the laboratory, the true test of implementation for such systems would be within a clinical environment and through pragmatic trials (50), or n-of-1 trials (51), which are more suitable for the highly personalized nature of intervention that is needed for the individuals with PD. Each of these trial types is designed to determine the effectiveness of an intervention, with pragmatic trials focusing on effectiveness in the context of routine clinical practice (50) while n-of-1 trials seek to evaluate the effectiveness of an intervention that is personalized to individual patients (51). Either of these trial types or a more conventional randomized trial is a feasible next step that we could perform at scale due to the following three facilitators: (a) our system hardware is consumer-grade and relatively affordable; (b) we have designed the application to have several features that can be individualized to the patient such as the word difficulty, time allotted to solve the puzzle, and the height and spacing of solution letters; and (c) allocating space that ideally allows for leaving the tracking cameras in place and demarcating the walkable volume on the floor with tape or paint is likely feasible in physical therapy clinics. Subsequent tests of effectiveness would require a systematic process to determine how best to structure features of the training environment, the necessary duration and frequency of training to achieve a clinically meaningful outcome, and the characteristics of patients who would best benefit from the intervention. Clinics would need to train therapists to use the system, which is not very difficult, and to determine the target population and adapt accordingly. For example, using it with patients at risk for falling would require the use of a safety harness, and using it with seated patients would require some tailoring of the features available within our game. Conducting a pragmatic trial would require that participants be willing to participate in the research study beyond their usual allocated PT time. However, the current game could replace up to 2 weeks of usual PT sessions. Greater use would require adding more depth to the current game, or new games that satisfy other PT needs. Ultimately, we will also need to consider the path to market and long-term viability of our application and the hardware on which it is used. The two paths to market include either commercialization by an entity who would sell the software and provide support to end users or provision of the software for free in an open-source format that is freely available and can be modified by the community (52). Regardless of the chosen path to market, the long-term success of interactive applications for health requires that we develop software that can be adapted to and implemented on both currently available and future devices for virtual and augmented reality.

## Data Availability Statement

The raw data supporting the conclusions of this article will be made available by the authors, without undue reservation.

## Ethics Statement

The studies involving human participants were reviewed and approved by The Institutional Review Board at the University of Southern California. The patients/participants provided their written informed consent to participate in this study.

## Author Contributions

JF, MG, VL, AK, and BF contributed to conception and design of the study. JF, SJ, and AK collected the data. JF, MG, SJ, AK, and BF contributed to the analysis and data visualization. JF, MG, SJ, AK, and BF wrote the first draft of the manuscript. All authors contributed to manuscript revision, read, and approved the submitted version.

## Conflict of Interest

The authors declare that the research was conducted in the absence of any commercial or financial relationships that could be construed as a potential conflict of interest.

## Abbreviations

IMI: intrinsic motivation inventory
ITC-SOPI: independent television commission sense of presence inventory
MDS-UPDRS: movement disorder society–unified Parkinson's disease rating scale
SSQ: simulator sickness questionnaire
SUS: system usability scale
VR: virtual reality.
